# Data Access Committees

**DOI:** 10.1186/s12910-020-0453-z

**Published:** 2020-02-03

**Authors:** Phaik Yeong Cheah, Jan Piasecki

**Affiliations:** 1 Mahidol Oxford Tropical Medicine Research Unit (MORU), Faculty of Tropical Medicine, Mahidol University, 420/6 Rajvithi Road, Bangkok, Thailand; 2 Centre for Tropical Medicine and Global Health, Nuffield Department of Clinical Medicine, University of Oxford, Old Road Campus, Roosevelt Drive, Oxford, UK; 3 The Ethox Centre, Nuffield Department of Population Health, University of Oxford, Oxford, UK; 4 Department of Philosophy and Bioethics, Faculty of Health Sciences, Jagiellonian University Medical College, ul. Michalowskiego 12, Krakow, Poland

**Keywords:** Data Access Committee, Data sharing, Ethics, Research ethics, Research ethics committees

## Abstract

**Background:**

Sharing de-identified individual-level health research data is widely promoted and has many potential benefits. However there are also some potential harms, such as misuse of data and breach of participant confidentiality. One way to promote the benefits of sharing while ameliorating its potential harms is through the adoption of a managed access approach where data requests are channeled through a Data Access Committee (DAC), rather than making data openly available without restrictions. A DAC, whether a formal or informal group of individuals, has the responsibility of reviewing and assessing data access requests. Many individual groups, consortiums, institutional and independent DACs have been established but there is currently no widely accepted framework for their organization and function.

**Main text:**

We propose that DACs, should have the role of both promotion of data sharing and protection of data subjects, their communities, data producers, their institutions and the scientific enterprise. We suggest that data access should be granted by DACs as long as the data reuse has potential social value and provided there is low risk of foreseeable harms. To promote data sharing and to motivate data producers, DACs should encourage secondary uses that are consistent with the interests of data producers and their own institutions. Given the suggested roles of DACs, there should be transparent, simple and clear application procedures for data access. The approach to review of applications should be proportionate to the potential risks involved. DACs should be established within institutional and legal frameworks with clear lines of accountability, terms of reference and membership. We suggest that DACs should not be modelled after research ethics committees (RECs) because their functions and goals of review are different from those of RECs. DAC reviews should be guided by the principles of public health ethics instead of research ethics.

**Conclusions:**

In this paper we have suggested a framework under which DACs should operate, how they should be organised, and how to constitute them.

## Background

Expectation from health research funders, regulatory agencies, and journals for greater sharing of de-identified individual-level health research data is now increasing [1–5], but the volume of data shared remains low [6]. Arguments in favour of data sharing include maximising the utility of the data, improving research transparency and allowing confirmation of the interpretation of results, with the overall goal of improving science and health [1, 2, 7, 8]. However, many have cautioned that there are potential harms with regards to data sharing, such as misuse of data, breaching participant confidentiality, group harms of discrimination and stigmatization, as well as exacerbating existing inequalities between researchers in low- and high-income settings [2, 9, 10].

There are ongoing discussions on how best to approach data sharing. The landscape of research ethics is evolving and after the eras of researcher and regulatory paternalism [11], a new paradigm shift is being discussed: an ethical framework for learning healthcare systems [12]. This paradigm brings a few new ideas. It puts into question the difference between research and routine clinical practice: new information technologies alter the nature of medical practice, which has recently become a learning activity where its goals are not limited to benefiting an individual patient, but also embraces the generation of knowledge. The main aim of an ethical framework for learning is not to protect individuals, but to promote the common good of an efficient and safe healthcare system. Therefore it has been argued that an ethical framework for learning healthcare systems is public health ethics [13]. By the same token, it has been suggested that data sharing should be governed by the principles of public health ethics, rather than research ethics [14]. This is because public health ethics focuses on public benefit, proportionality, accountability, equity and trust while research ethics tends to focus on consent and individual interests [14]. There are significant similarities between public health activities, epidemiological research and data sharing in terms of goals, benefits and risks associated with these activities. Therefore in this paper we defend a position that data sharing should be guided by the principles of public health ethics than by those of research ethics. We provide reasons for this position in the later part of the paper.

Health researchers and ethicists have proposed that one way to promote potential benefits of data sharing and ameliorate its potential harms would be through the adoption of a managed access approach where requests are channeled through a Data Access Committee (DAC), rather than making data openly available without restrictions [15]. DACs, a formal or informal group of individuals who have the responsibility of reviewing and assessing data access requests [15], have only been developed relatively recently. Many group, consortia, institutional and independent DACs have been set up but there is currently no widely accepted framework under which DACs operate.

This paper aims to suggest a framework for DACs and to advance discussions on what the functions of these DAC should be, how they should be organised, and how to constitute them. While many previous discussions have centred around genomic data, our paper discusses DACs which operate as custodians of all types of health data generated from public funded health research. The sharing of data collected in a clinical context or health administrative data, insurance data is beyond the scope of this paper.

We propose that DACs should have the role of both promotion of data sharing and protection of data subjects, their communities, data producers and their institutions. With these roles in mind, we then discuss the organisation and composition of a DAC. We suggest that DACs should not be modelled after research ethics committees (RECs) and give reasons why. In addition, we suggest that DAC reviews should be guided by the principles of public health ethics instead of research ethics.

## Main text

### Functions of Data Access Committees

#### Promotion of data sharing in the interest of science, data producers, data subjects and their communities

We think that given DACs central role in data sharing, they have an important role in promoting data sharing. The primary question for a DAC should be whether the nature, degree, and likelihood of possible public benefits from the data reuse outweigh the nature, degree and likelihood of possible harms to the data subjects, relevant communities, or other stakeholders e.g. primary researchers, their institutions, or countries.

The features of ethical primary research studies are well established. Human subject research guidelines identify that the first question for any research should be whether the proposal is likely to generate scientifically sound, socially valuable knowledge [7, 16]. However, unlike primary research, data access should be granted as long as the data reuse fulfils the criterion of having even a minimal social value [7], and minimal risk to data subjects and their communities (we discuss risks to primary researchers and their institutions later in the paper). A critical and skeptical approach to existing knowledge is an important element of the scientific enterprise. Therefore, reuse of data does not always have to generate new knowledge; it is of significant social value when it verifies results of previous research, for example. Moreover, the analysis of already existing data could spur new scientific hypothesis and guide new research projects [8].

Although data research carries risks, it is not equivalent to enrolling subjects in new clinical or observational research studies. The nature and magnitude of risks arising from secondary data use is different from the nature and magnitude of risks of original studies. This fact is reflected in many existing international and national regulations. For instance, in the European Union General Data Protection Regulation (GDPR) (Art 5(1)(b), Art 89), data-driven health research is given a special derogation and, if researchers demonstrate that a study is “in the public interest” a REC may grant the study a waiver of informed consent [17, 18]. Similar standards are present in the United States Common Rule (45 CFR §46.116, 45 CFR §164.512) and in the Health Insurance Portability and Accountability Act (HIPAA) [19], both regulations grant several derogation for data-driven research involving public benefit, posing minimal risk and allow access to identifiable health information [20, 21].

For data sharing to be successful, there must be a win-win situation for both data producers and secondary data users, their wider teams and institutions. DACs should encourage secondary uses that promote the interests of data producers, such as research that contributes to the goals of their institution [22]. For example, the DAC for an institution with the goal to improve the treatment of malaria should encourage data reuse that ultimately contributes to malaria treatment improvement. However, it does not mean that data should not be shared if the objective of the secondary use conflicts with institutional priorities.

In addition, researchers could reap greater benefits from their data when others are involved in the secondary data analysis (e.g. mathematical modellers). Data sharing can increase scientific productivity. In most cases, it is better to collaborate with others than doing it alone, or planning to conduct the secondary analyses but never getting to it. These extra outputs that arise as a direct result of data sharing will help maximise the utility and cost-effectiveness of studies and in turn increase the overall output and visibility of the institution and its members. This serves as a powerful internal incentive for data producers. If they benefit from data sharing, then data sharing would not be perceived by researchers as another box to tick or an obligation imposed by funders and journals.

Many secondary analyses involve research in the same disease or topic, which could directly benefit the data subjects and their communities, but there are also many secondary data uses that will not have these direct benefits. Some data research will confirm existing results, and others will advance the knowledge of the disease or topic thereby potentially benefiting future patients in downstream research. Other data uses could be for teaching purposes or for shaping a new project. Irrespective of how the data are used, provided that there are some benefits to society, we are respecting the altruism and commitment of data subjects by reusing their data.

#### Protecting data subjects, their communities, data producers, their institutions and the scientific enterprise

Data sharing has triggered concerns for the privacy of the data subjects. Data scientists have proved on multiple occasions that datasets that were thought to be anonymized i.e. “personally identifiable information (PII) is irreversibly altered in such a way that a PII principal can no longer be identified directly or indirectly” [23], could be linked with other public health data to identify the specific data subject [24, 25]. However, there is scarce evidence that these individual data breaches have resulted in individual harms [26]. In fact, many of the potential harms apply to the data community, rather than individuals. These potential harms apply even when data are anonymized, because of potential group harms be it by geography, disease or ethnicity. Some have voiced concerns over potential harmful uses that could result from stigma and discriminatory uses by employers or insurance companies [14, 27]. Hence, although the primary role is to promote data sharing, DACs should also be aware of potential group harms and when such risks are more than minimal, data reuse should not be allowed. These potential group harms sometimes depend on the economic and cultural context of data subjects. They are more worrying in places that have a history of targeting and discriminating against minorities, countries that do not provide their citizens with universal access to healthcare and where access to healthcare depends on private insurance or ability to pay. We recognize that that DACs do not necessarily know what these group harms will be. There will be risks of underestimating or misidentifying potential harms to data subjects and their communities. These risks can be minimized by careful engagement with research communities during primary research [28, 29], e.g. consultation with community leaders and community advisory boards [30–32].

Protection of data subjects also entails protecting their rights. DACs should make sure that the shared data do not contain any personally identifiable information, and that data will be used within the scope of broad consent provided by subjects. In the case of old datasets where broad consent for sharing had not been obtained, DACs should adhere to the criteria set out by CIOMS 2016: the secondary use offer important otherwise unobtainable information, has social value, and poses minimal risks to the subjects, and that it would be impractical or prohibitively expensive to contact subjects for their consent for secondary use [7].

There have also been worries that data sharing can disadvantage data producers and potentially dis-incentivise primary research [10]. This would be detrimental to the research enterprise and scientific progress. In order to prevent this, DACs should provide guidelines, within constraints of funder and regulatory requirements, on when specific conditions of access should be put in place. These could include recognition requirements such as authorships, acknowledgements or standard citations. In some cases, collaborations may be necessary, especially where interpretation of the data requires the experience of the primary researchers and an in-depth understanding of the context. In addition, an institution may have exclusive access periods, requirements for benefit sharing, preferential access provisions (e.g. to collaborators) and embargo periods. In addition, DACs should mandate when formal data access agreements to specify terms of access should be signed and if a cost recovery or cost sharing mechanisms should be put in place.

### Establishment, composition and pocedures of a Data Access Committee

#### Establishment

The influential Council for International Organization of Medical Sciences (CIOMS) 2016 guidelines recommend that “when data are stored, institutions must have a governance system to obtain authorisation for these data in research” [7]. In addition, the guidelines state that “the ethical acceptability of broad informed consent relies on proper governance”. Governance of data, which includes data access mechanisms is ideally outlined within the institutional, group or departmental data sharing policy. We suggest that DACs should be established within institutional and legal frameworks with clear lines of accountability, terms of reference and membership.

Some suggest that DACs should be independent of the institution to avoid any conflicts of interest. Indeed many independent DACs exist for this reason such as the MalariaGEN and Managing Ethico-social Technical and Administrative DACs [33, 34]. To motivate data sharing, we must recognise that sharing data might reveal sensitive information not only about data subjects but also about researchers, healthcare providers and/or their institutions which might cause harm or embarrasment [35]. However, the argument for DACs to be institutional, instead of completely independent is ultimately a practical one. If institutions reserve the final authority regarding data sharing decisions, then they will be more willing to share their data. Institutions are the custodians of data and they should act on behalf of research participants, who have consented to broad research reuse of their data. Institutional DACs are then accountable to both their home institution and their research participants. It is unclear who independent DACs are accountable to.

#### Composition

In order to fulfill the functions of a DAC as described in the previous section, a DAC should consist of a reasonable number of members, each covering multiple relevant areas of expertise. For DACs of large research groups, departments or institutions, ideally there should be members representing senior management, data management, ethics, relevant research areas and potentially a data sharing advocate. It is also desirable to have independent members to address the issue of conflicts of interest and to prevent “data hoarding” by researchers internal to a study.

The CIOMS guidelines state that DAC should have “representation from the original setting” [7]. In the context of large clinical studies or institutional DACs that review multiple studies, it is not feasible to have “representatives of the original setting” serve as members on the DAC. However, having members who are familiar with the context or contexts where the research is conducted is necessary. Some data reuse may require consultation with study investigators, country or community representatives. DACs can also consult on an ad-hoc basis with people familiar with the community or data subjects where necessary.

#### Application procedures

In order to promote data sharing, there should be transparent, consistent, simple and clear procedures for data request and data access. The approach to the review of applications should be proportionate to the potential risks involved and streamlined because DACs approve or disapprove data already collected, rather than new research studies. Reviews should be guided by the data sharing policies of institutions or pre-agreed terms in the case of independent DACs; and DAC reviews, as we argue in the next section, should not be guided by review criteria adopted by RECs. Elements of a DAC review should include among other things who is applying, what are the objectives of data reuse, exactly what data are requested, anticipated benefits and potential risks.

### Why Data Access Committees should not be modelled after research ethics committees?

One can ask a question, should DACs be modelled after the RECs (in the US context, known as Institutional Review Boards) system that reviews new human subject research? There are a few organizational and ethical reasons for devising a different *modus operandi* for DACs and these reasons pertain to: organizational culture; goals of review; ethical framework of review; accountability to the host institution.

#### Organizational culture

Conceptual analyses and empirical studies reveal that there is an inbuilt adversarial relationship within the system of a research ethics review [36–38]. If the main function of RECs is to protect research subjects, then there is an implicit assumption that research poses risks, and puts the burden of proof that this is not the case on researchers. As such, researchers do not perceive RECs members as ethics advisors, but as judges and punishers: researchers have to prove they have good intentions [37].

Regulations give RECs a function of protecting research participants by ensuring that they are provided with proper information and that research projects have a favourable risk-benefit ratio. However, there is no a universal standard of how exactly the RECs should function. Some suggest that RECs should check, if research protocols are consistent with certain ethical or legal codes rather than with abstract ethical principles [39]; while others argue that RECs should “perform ethically informed code consistency review” [40]. As result of these disagreements, over time the function of protection has evolved and RECs have acquired new functions, some of them intended, some were a consequence of institutional logic and gradual legalisation of ethics reviews [36]. Hence RECs guard research integrity and quality by filtering “bad science” projects, and protect community by screening wasteful and dangerous studies [37]. In this sense, members of RECs often perceive themselves as acting on behalf of communities. Moreover, when one looks at a REC from a wider perspective and takes into consideration the fact that their function is grounded in democratically enacted law, these institutions could also have a function of providing political and ethical legitimacy either to a single study or to biomedical research in general [41]. Proliferation of functions coupled with inconsistencies among RECs in multicenter studies have resulted in growing bureaucratic and financial burden on researchers. This has led to a recognition that an ethics review might be in certain instances an overprotection of research subjects that leads to underprotection [12, 42]. A REC delaying a low-risk study, may at the same time expose patients to substantial risks, for instance in a situation when a post-marketing study would provide evidence for serious adverse effects of already approved drugs [43]. This recognition has been reflected in the regulations that do not require a full-ethics review of low-risk studies and the introduction of central reviews for multicenter studies.

Although an adversarial relationship between RECs and researchers may be legitimate in riskier clinical trials, an adversarial relationship between DACs and primary researchers or secondary users is inconsistent with the desired goals and functions of DACs. As we have argued, DACs should promote reuse of research data. DACs should be part of new research culture that helps in promoting scientific progress. Therefore, the main function of DACs is not defined in adversarial terms of “protection”, and DACs should be conceived as an institution’s tool for realising its goals. Those who apply for access to data should not be perceived as a potential danger, but as potential collaborators.

#### Goals of review

A REC comprising of researchers, lawyers, ethicists, nurses, patient representatives and community representatives is intended to bring a diverse perspective on scientific and ethical aspects of a study involving human beings. The goals of an ethics review are to discern those aspects of a study that were overlooked or misjudged by a researcher whose perspective might be skewed by conflicting interests, and to ensure that the research complies with specific laws and research ethics guidelines.

A REC may work in accordance with either a panel of professionals or a jury board [44]. In the case of a professional panel, a REC adjudicates from the position of a professional and objective judge; in the case of a jury board, a REC makes its decision from the point of view of a reasonable layperson. The main goal of ethics review is to protect research participants, and although it is the principal investigator and sponsor who bear the ultimate responsibility for the well-being of research subjects, RECs are at least morally responsible too.

RECs review new research studies and any secondary data research that requires ethical approval which can differ from jurisdiction to jurisdiction. DACs review data access requests for secondary uses. These uses may be for secondary data research but could also be for teaching purposes, to confirm the findings of the original analyses or other purposes. DACs roles should not include full review of the secondary research such as methodology of the secondary research and the statistical approaches. That is the job of RECs.

The secondary use of data already collected differs significantly from conducting clinical research. A secondary data user does not interact with research subjects; data research does not require additional diagnostic tests or examinations and the possible risks to an individual are often limited to privacy breaches and group harms. DACs have different objectives from RECs. DACs are custodians of research data, but this function cannot be understood as a protection from intruders, who may want to have a peek into their treasure trove, but promoters of the beneficial use of data. Institutional DACs should also review the consistency of data use with institutional data sharing policies. The goal of this review should include maximising data research utility either by confirming previously tested results or generating new data, and assessing if there are any potential harms to all relevant stakeholders.

#### Ethical framework for review

In its origins, research ethics has been informed by the scandals and tragedies of research, such as atrocities of Nazi doctors, the Willowbrook School Experiment, the Jewish Chronic Disease Hospital and the Tuskegee syphilis studies [11]. Due to these historical events, the main goal of research ethics framework and resulting guidance documents was protection of individual research participants. Moreover, those who were conducting medical research were physicians themselves, whose professional identity was intrinsically connected with an obligation to protect and promote individual patient interests. This is why many research ethics guidelines, such as the Oviedo Convention [45] and the Declaration of Helsinki [16], contain some version of the principle of precedence of individual interests, for example, “the interests of an individual should prevail over the sole interest of society or science” [46]. However, it has become clear recently, that the principles of research ethics cannot be universalised, and not all kinds of research can be held to the same ethical and procedural standards. For instance, an ethical review and an informed consent procedure may seriously impede multicentre epidemiological studies [47]. The goals of epidemiology and public health are different than those of clinical research. The main focus of epidemiology and public health is not an individual patient, but promotion of population health [48]. Moreover, in public health research it is much more difficult to distinguish research from routine clinical practice [13, 49, 50].

Similar problems of inadequacy of the research ethics approach to multicenter data-driven research has been recently discussed in regards to learning healthcare systems (LHS), in which conducting research is embedded into healthcare practice [12, 42]. The learning process is driven by data that are produced in healthcare practice and then collected and analysed in a search for generalisable knowledge. Efficiency of LHS requires a different ethical approach. Here again protection of an individual is not a priority, since an individual patient is not exposed to risks other than those inherently associated with healthcare practice. The conceptual and ethical framework for LHS also applies to principles of public health ethics rather than research ethics - to weigh public benefits against possible infringements of individual rights [12, 13, 48].

Epidemiology, public health research, and LHSs have a few common characteristics: the benefits and risks pertain to groups rather than to individuals; in many cases the research activity consists of collecting and analyzing vast volumes of data; research ethics standards (e.g. full-informed consent, full-ethics review) are not feasible and can either hamper research or put extra bureaucratic burden on researchers. In all three cases it is clear that the public health ethics approach is applied. Data sharing, at least in two important respects, has similar characteristics to epidemiology, LHS and public health research: the benefits and risks of data sharing pertain to groups rather than to individuals, and data sharing is about accessing and processing vast volumes of data and there are minimal additional risks to a data subject. Taken together, the more suitable ethical approach to data sharing should be that of public health ethics instead of research ethics [14].

#### Accountability to host institution

There are at least two models of a REC: independent bodies established by private or public actors, or RECs that are established by research institutions such as universities. Both models provide independent review of research studies. Independence does not mean that there is no institutional links between a REC and a research institution, but it means that the research institution does not influence the workings of its REC which should be independent in its judgments of the ethical standards of studies. Importantly, RECs do not implement any institutional research policies nor are they tasked to promote research.

We think that DACs should play a central role in implementing institutional policies on data sharing. This is yet another reason for DACs to be institutional rather than independent. The task of a DAC is to balance the goals and policies of its institutions, the goals and interests of those who apply for data and the public good. RECs protect research subjects by applying ethical principles and rules of law; DACs should promote data sharing while mitigating any potential risks, and should be a mechanism to implement institutional data sharing policies. While it is challenging to evaluate the efficacy and efficiency of RECs, it is possible to evaluate those of a DAC, that is by measuring the realisation of data sharing goals and policies.

### Strengths and limitations

Our normative proposal is supported by experience of establishing and coordinating the Mahidol Oxford Tropical Medicine Research Unit (MORU) DAC, which has reviewed over 40 applications since its establishment in January 2016 [51–53]. The MORU DAC has reviewed many types of data requests including data in real time from an ongoing clinical study, from historical trials done without participant consent for data sharing, and from pharmaceutical companies for data from trials conducted in low-resource settings for registering products in developed countries [52, 54].

Our suggestions are primarily focused on DACs of publicly funded large research groups, departments or institutions conducting clinical research. We acknowledge that some research groups may be too small and may not have the resources or skills to establish and run their own DACs. Efforts are underway to provide support for research groups in low-resource settings to establish their own data sharing policies and DACs. We think that future empirical research is needed to verify the feasibility and efficacy our suggestions, and compare them with other models of DACs such as DACs established to review requests for health system data.

## Conclusions

In this paper we have suggested a framework for the functions and establishment of DACs and demonstrated that sharing de-identified health data should be governed by a different conceptual and ethical framework than clinical research involving human beings. DACs should promote the beneficial use of data, while mitigating any potential harms in line with the ethical framework for public health instead of research ethics. We have also argued that the system of ethics review as it is operated by RECs is not suitable for the realizing the ideals of data sharing and therefore should not be a model for DACs.

## Data Availability

Not applicable.

## Abbreviations

DAC: Data Access Committee
LHS: Learning healthcare system
MORU: Mahidol Oxford Tropical Medicine Research Unit
REC: Research ethics committee
